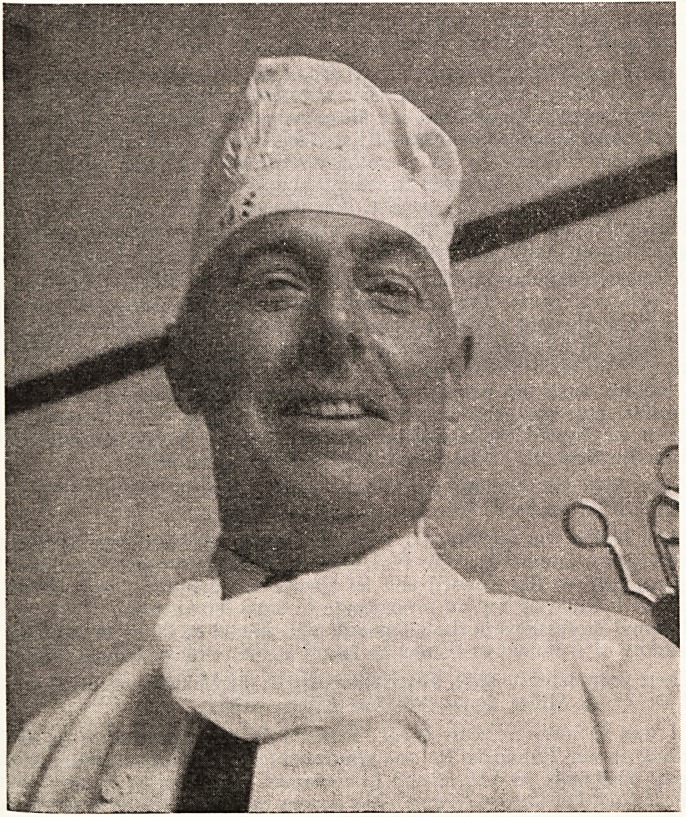# D. G. C. Tasker

**Published:** 1968-04

**Authors:** 


### OBITUARY

### D. G. C. TASKER, B.SC., M.S., F.R.C.S.

Mr. Douglas George Clutson Tasker, Consultant Surgeon to the United Bristol Hospitals died after a short illness at his home on the 18th December 1967 at the age of 74, having given 40 years of faithful service to hospitals in the Bristol Clinical Area.

Born at Weston-super-Mare on the 25th May 1893, where his father was in business, he received his early education at Brynmellan School. From here in 1908 he entered the Medical School of the University College of Bristol, the year prior to the University receiving its charter. The Medical School was then situated in Tyndalls Park. A natural athlete, he played football, tennis and represented Gloucestershire at badminton. His elder brother, who also qualified in medicine, was a brilliant amateur footballer and played for the Bristol City at a time when the Club was in the first division; he lost his life in the first world war.

Douglas Tasker started his pre-clinical subjects in medicine and graduated B.Sc. at the University with first class honours in biochemistry at the age of 20, and it is possible to imagine that he might easily have made this subject his future career. While he continued with his medical studies he joined the 6th Battalion Territorial Worcestershire Regiment. 1915 saw him serving in France as a combatant Officer, and during this period of service he was awarded the Croix de Guerre. Later that year he was ordered home to complete his medical studies and this necessitated him resigning his commission. Qualifying M.B., B.S. as an external student of London University he was commissioned in the R.A.M.C. and served for the duration in Salonika.

Returning to Bristol in 1918 he was appointed House Surgeon at the Bristol General Hospital and subsequently in 1920 Senior Resident Officer. Whilst holding this latter post he was successful in passing both his M.S. Lond. and F.R.C.S. During these years his surgical work was guided by the following surgeons of the staff of the hospital, Messrs. J. Lacy Firth, C. A. Morton, R. G. P. Lansdown, E. W. Hey-Groves (the original Editor of the British Journal of Surgery) and the assistant surgeons were Messrs. C. A. Moore and Duncan Wood. Appointed Assistant Surgeon in 1923 and full surgeon in 1932, he held this post until with the amalgamation of the Bristol General Hospital and Bristol Royal Infirmary in 1939 he became Honorary Surgeon to the Bristol Royal Hospital. After 1948 he became Consultant Surgeon to the United Bristol Hospitals, continuing his work at the Royal Infirmary branch until 1958 when he retired.

In his early years other appointments rapidly came his way. He held appointments at Cossham Hospital, Clevedon and Berkeley Cottage Hospitals and the Bristol Mental Hospital.

A first class general surgeon with a wide knoweldge, he had the great gift of simplifying the principles of surgery so that his students never forgot his teaching. His operative work was precise and unhurried and only the clock showed the speed with which an operative procedure was completed. He never seemed to be in difficulties and he had the same gift of simplifying both clinical and operative surgery when teaching at Registrar level. His senior resident officers and registrars owe much to these clear principles he imparted. To those who remember his out-patient clinics and ward rounds, his opening question to the patient was always " What do you find the matter ? " His gall bladder and biliary surgery was faultless and he always enjoyed at least one or more impossibly large or recurrent hernia which he usually repaired with a Gallie fascial graft. He pioneered cranial surgery at the General Hospital and his old house surgeons remember the meticulous way in which these procedures took place. Always operating on a Sunday morning so that the pressure of time and other operating lists was excluded, the physician in charge of the case, usually Dr. H. H. Carleton, but sometimes another member of the medical consulting staff, assisted at the operation. In the late 1930s when one of the younger Bristol surgeons commenced to specialise in this branch of surgery, Mr. Tasker handed all this work over, believing it was no longer the province of a general surgeon to continue in this field.

An invitation to accompany him to one of his Cottage hospitals was always a pleasure : the journey consisted of a succession of stories of both recent and past experiences. The visit to the Hospital always in consultation with the general practitioner who presented the case, included an out-patient session, ward round, followed by an operating list of selected cases again with the general practitioner concerned assisting. Always a great supporter of contact between the consultant and general practitioner, it was while he was senior resident officer in the 1920s that the system of discharge letters from hospital to general practitioner was instituted.

A founder member of the Surgical Travellers Club which was formed in 1927 he was a regular attender at their meeting, both in Europe and at home, being highly thought of both on professional and personal grounds. During his early years as an honorary surgeon he developed an interest in medico-legal work and so much was his opinion valued that subsequently it was necessary for him to limit the number of cases he was able to report on. In the immediate post-war years he was appointed Chairman of the Medical Committe of the Royal Hospital and his great knowledge of hospital administration and the implications of the Health Service was invaluable.

From 1946 he gave his experience to the Medical Defence Union, and Dr. Phillip H. Addison, Secretary, writes as follows :

" Mr. Tasker was appointed a Vice-President in 1946 and was Chairman of the Executive Committee from 1951 to 1956. His wide surgical experience was of great help to the Council and he always felt that the Union should do whatever it could by way of propaganda to draw the attention of the medical and dental professions to the safeguards that should be taken to minimise the possibility of a patient sustaining an injury as the result of human fallibility."

Three years later in 1949, he gave his help to the Ministry of Social Security and Dr. G. A. Miller, Senior Medical Officer, writes :

" I was so sorry to hear of Mr. Tasker's death so soon after he finished working on our Tribunals. He was appointed to our Industrial Injury Medical Appeal Tribunals on 4.10.48 and he remained a member of those Tribunals until 25.5.65. On the 1st August 1958 he was appointed to the War Pensions, Pensions Appeal Tribunals, and in July 1959 he became Chairman of one of the Assessment Appeal Tribunals and did not retire from this until February 1966. You can appreciate that after this long period during which he helped us we were very sorry when he had to resign on age, since he was so very experienced and helpful on these Tribunals."

It is difficult to imagine that with so many commitments, especially in the latter years as Chairman of the Appeals Tribunals which necessitated travelling throughout the country, he had time to give his services elsewhere. However, in 1961 he was appointed Chairman to the Joint Registrar Appointments Committee and the Joint Advisory Committee on Senior Registrars (posts which he still held). These Committees whilst dealing with policy, matters on the training of Registrars and the review of their progress, was responsible for all appointments throughout the South West region, an area extending from Cheltenham in the north to Penzance in the south-west. Often meeting twice monthly the Appointments Committee might well continue all day and the Chairman remained as charming and understanding to the candidate at the end of such a long day as if it was the first interview in the morning. His great integrity and fairness was known to all who were invited to attend these meetings and serve under his chairmanship. Only three days prior to his death he sent his apologies to the Committee as he was not feeling well enough to chair the meeting that day.

His fondness for athletics was mentioned earlier, and in later years he turned his attention more to golf, but on retirement he added gardening to his interests, obtaining a great knowledge of the subject by study so that his green-house gave him as much pleasure as his sweet peas which were of exhibition standard. A great lover of music, which he enjoyed rather more in his home than by visiting concert halls.

He will be greatly missed by the many. To his widow, Mrs. Tasker, and his son Alan we offer our deepest sympathy in their sad bereavement.

## Figures and Tables

**Figure f1:**